# Risk Assessment in Food Safety

**DOI:** 10.3390/foods15071190

**Published:** 2026-04-01

**Authors:** Adriana Pavesi Arisseto Bragotto

**Affiliations:** Departamento de Ciência de Alimentos e Nutrição, Faculdade de Engenharia de Alimentos, Universidade Estadual de Campinas (UNICAMP), Campinas 13083-862, SP, Brazil; pavesi@unicamp.br; Tel.: +55-19-3521-0066

Food safety is a fundamental pillar of public health and a prerequisite for food security, as safe food is essential to ensure that diets support both human health and well-being [1]. In an increasingly globalized and complex food system, safeguarding food safety has become more challenging due to extended supply chains, technological change, evolving diets, and emerging pressures such as climate change and food system transformation. While food safety has long been a concern, these interconnected drivers have increased the complexity and diversity of potential hazards in food, including microbiological and physical agents, chemical contaminants, food additives, residues, and allergens [2]. Such hazards may compromise food safety and, consequently, human health, highlighting the need for robust scientific approaches to identify, evaluate, and control them.

Within this context, a critical distinction must be made between hazards and risks. A hazard refers to any biological, chemical, or physical agent in food with the potential to cause adverse health effects, whereas risk reflects the likelihood and severity of such effects occurring under specific conditions of exposure to a hazard [3]. Thus, the mere presence of a hazard does not necessarily imply a significant risk. This distinction is essential for avoiding overly conservative or misleading conclusions and for enabling the prioritization of food safety issues based on their actual impact on public health.

Risk assessment has therefore been globally recognized as a key scientific tool to support decision-making in food safety. It encompasses four steps: hazard identification, hazard characterization, exposure assessment, and risk characterization [3]. In recent years, significant advances have been made in refining these approaches, including the adoption of probabilistic models to better capture variability and uncertainty [4], as well as the incorporation of concepts such as bioavailability, bioaccessibility, and cumulative exposure [5]. In parallel, New Approach Methodologies (NAMs), including in vitro systems, in silico models, and omics-based approaches, have emerged as promising tools to support hazard characterization and reduce reliance on traditional animal testing [6]. Despite these advances, important challenges remain, particularly in dealing with data gaps, emerging hazards, and the complexity of dietary exposure scenarios, reinforcing the need for continuous methodological development and interdisciplinary collaboration.

The contributions to this Special Issue reflect the diversity and evolution of research in risk assessment applied to food safety. The review by Pekmezci et al. [Contribution 1] provides a comprehensive overview of dietary chemical exposure and its implications for human health, setting the stage for the studies presented. Several contributions focus on exposure assessment, including the study by Stojanović et al. [Contribution 2] on dietary exposure to tropane alkaloids in cereal-based products in Serbia, the work by Ku et al. [Contribution 3] assessing food preservative intake among eating-out populations using a total diet study approach, and the study by Pôrto and Arisseto Bragotto [Contribution 4] estimating food color exposure in the Brazilian population based on national consumption data. Methodological advances are further illustrated by the study of Ma et al. [Contribution 5], which investigates the role of bioaccessibility in refining exposure assessment of pesticide residues. In addition, the work by Peycheva et al. [Contribution 6] presents a benefit–risk analysis of toxic and essential elements in fish, highlighting the importance of integrating both adverse and beneficial effects in food safety evaluations. Finally, Alewijn et al. [Contribution 7] analyze food fraud cases in the European Union, providing important insights into the relationship between fraud and food safety hazards. Collectively, these contributions emphasize the importance of realistic exposure assessment, context-specific data, and innovative methodologies to support more robust risk characterization. Ultimately, strengthening risk assessment is essential not only to protect public health but also to underpin resilient and sustainable food systems and ensure true food security.

In conclusion, advancing risk assessment in food safety requires not only methodological innovation but also the integration of multidisciplinary knowledge and context-specific data. As food systems continue to evolve, strengthening the scientific basis of risk assessment will be essential in order to address emerging challenges, reduce uncertainties, and support effective decision-making. The contributions to this Special Issue highlight both the progress achieved and the work that remains, reinforcing the central role of risk assessment in protecting public health and ensuring sustainable and safe food systems.

## Data Availability

Not applicable.
